# Effect of Combining Therapy with Traditional Chinese Medicine-Based Psychotherapy and Herbal Medicines in Women with Menopausal Syndrome: A Randomized Controlled Clinical Trial

**DOI:** 10.1155/2012/354145

**Published:** 2012-12-10

**Authors:** Hongyan Yang, Jing Yang, Zehuai Wen, Qinglin Zha, Guangning Nie, Xuchun Huang, Chunlin Zhang, Aiping Lu, Miao Jiang, Xiaoyun Wang

**Affiliations:** ^1^Department of Gynecology, The Second Affiliated Hospital, Guangzhou University of Traditional Medicine, Guangzhou 510120, China; ^2^Institute of Basic Research in Clinical Medicine, China Academy of Chinese Medical Sciences, Beijing 100700, China; ^3^School of Computer, Jiangxi University of Traditional Chinese Medicine, Nanchang 330004, China

## Abstract

This multicenter, randomized, controlled clinical study was designed to address the effectiveness of combined traditional-Chinese-medicine- (TCM-) based psychotherapy and Chinese herbal medicine (CHM) in the treatment of menopausal syndrome. Altogether 424 eligible women diagnosed as menopausal syndrome and categorized as Kidney-Yin/Kidney-Yang deficiency pattern in TCM were randomly assigned into 4 groups and accepted TCM-based psychotherapy (PSY), CHM, PSY + CHM, or placebo therapies, respectively, for 12 weeks, and another 12 weeks were taken as the followup. Kupperman Index (KI) and the Menopause-Specific Quality of Life (MENQOL) with its four subscales (vasomotor, physical, psychosocial, and sexual) were employed for efficacy assessment. Results showed that 400 participants completed 12-week treatment, of which 380 finished the record of KI and MENQOF at week 24. The average adjusted number of KI score decreased between baseline and 12 weeks in all groups. Statistically significant differences were detected in the average adjusted change between the PSY + CHM group and placebo at overall time points (*P* < 0.05). No severe adverse events occurred in each group and no significant differences were indicated between any of the three groups and placebo in adverse event proportion. We concluded that TCM psychotherapy combined with CHM has a favorable outcome in treating menopausal syndrome.

## 1. Introduction

Menopause is the phase of life when a woman transitions, both mentally and physically, from sexual maturity to old age. In this phase, many females suffer from a considerable variety of symptoms; these can include hot flashes, night sweats, menstrual irregularities, vaginal dryness, depression, nervous tension, palpitations, and others [1]. Such symptoms are frequently considered part of menopausal syndrome. Approximately 20–75% of all perimenopausal women have to seek medical consultation or treatment and 10% of postmenopausal American women are currently using estrogen replacement therapy [2, 3]. 

The symptoms of menopausal syndromes are diverse. Researchers have documented the occurrence of certain psychological and somatic symptoms in specific clusters [4]. Some of these symptom clusters, such as vasomotor symptoms and sexual difficulties, were best predicted solely by menopausal status [1] and can be more effectively controlled by hormonal interventions than placebo [5]. Others, such as psychological and somatic symptoms, were more clearly associated with psychosocial factors [4]. Women who seek medical help for menopausal problems tend to report more physical and psychological problems in general [6], and women with more negative attitudes towards menopause in general report more symptoms during the menopausal transition [7]. These symptoms can include mental disorders, such as depression, which is a significant psychological symptom present in menopausal syndrome. Depression in particular has a proven association with perimenopausal status [5, 8]. Thus, classic hormone replacement therapy (HRT) cannot relieve all of the diverse symptoms that may be present, and better intervention is needed for relieving menopausal symptoms. 

It is known that the most relevant factors influencing a woman's quality of life during the menopausal transition appear to be her previous emotional and physical health, social situation, experience of stressful life events (particularly bereavements and separations), and beliefs regarding menopause [6]; treatment, as a result, should be planned with consideration given to those aspects. With this in mind, the inclusion of clinical psychologists and counselors as part of a therapeutic team is recommended [6]. A clinical pilot study has shown that cognitive-behavioral techniques can reduce symptoms (including depression and anxiety) of menopausal syndromes compared to a control group [9]. A randomized controlled trial documented that Chinese medicine therapy combined with psychological intervention could not only improve nervous symptoms but also regulate the blood levels of lipids and sex hormones in patients diagnosed with peri-menopausal syndrome complicated with hyperlipidemia [10]; however, the clinical trial did not utilize a placebo control. Thus, solid evidence-based clinical studies involving a combination of medicine and psychological intervention are needed to assist clinicians in the management of menopausal syndromes. 

Traditional-Chinese-medicine-(TCM-) based psychotherapy refers to any form of therapeutic interaction or treatment that aims to increase the individual's sense of well-being. It is based on TCM theory and experiences, which are essential to TCM therapeutics, and it has been widely used in the treatment of many diseases. Favorable outcomes have been achieved, especially for diseases associated with psychological symptoms, such as menopausal syndrome [11]. In the present study, a randomized placebo-controlled clinical trial was conducted to address the efficacy of combined TCM-based psychotherapy and CHM in the treatment of menopausal syndrome. 

## 2. Methods 

### 2.1. Study Design

This 6-month-long randomized controlled trial was conducted at 7 clinical centers (Guangdong Province TCM Hospital, Beijing Xiyuan Hospital, The First Hospital Affiliated to Guangzhou University of TCM, Affiliated Hospital to Hubei College of TCM, Longhua Hospital Affiliated to Shanghai University of TCM, Affiliated Hospital to Chengdu University of TCM, and Tianjin institute of TCM) in China between June 2002 and October 2007. The study was conducted according to the principles of the Declaration of Helsinki after obtaining approval from the Ethics Committee of Second Affiliated Hospital of Guangzhou University of Traditional Medicine (Approval document no. B2002-03-1). Written informed consent was obtained from all participants. Because the trial included TCM-based psychotherapy, a blinded design could not be conducted. The efficacy was evaluated by a third party who was unaware of the interventions. 

### 2.2. Participants

#### 2.2.1. Eligibility Criteria

Patients were between 45 and 55 years of age at the time of informed consent and had been diagnosed with menopausal syndrome by 2 separate gynecological physicians. Furthermore, they met the TCM-pattern diagnosis of kidney deficiency. 

According to the New Drug Chinese Treatment of Female Menopausal Syndrome Clinical Research Guidelines (internal document: published by Ministry of Health P. R. China in 1997) criteria for menopausal syndrome, all patients should be at 45 to 55 years old and showing symptoms of menstruation disorder or amenorrhea, accompanied by either hot flashes and sweat or cold extremities. 

The TCM kidney deficiency pattern was divided into Kidney-Yin deficiency or Kidney-Yang deficiency, and the criteria for the two patterns were identified according to the Criteria in TCM Symptoms of Menopausal Syndrome [13]. All patients with all of the major symptoms and at least 2 of the secondary symptoms can be identified with either a Kidney-Yin or Kidney-Yang deficiency. For a diagnosis of Kidney-Yin deficiency pattern, the major symptoms include hot flashes and sweat, and the secondary symptoms include vexation, irritability/restlessness, insomnia, dry mouth and constipation, red tongue, shortage of tongue coating, and fine and rapid pulse. For Kidney-Yang deficiency pattern, the major symptoms include cold extremities or alternatively a fear of cold or hot flashes, and the secondary symptoms include dark complexion, dizziness, soreness and weakness in the lower back and knees, nocturia, thin and pale menstrual bleeding, pale tongue, thin white tongue coating, thinness, weakness, and sunken pulse. Two experienced TCM doctors with specific training for this study were responsible for the TCM pattern diagnosis in each study hospital. 

#### 2.2.2. Exclusion Criteria

All patients with bilateral oophorectomies, ovarian neoplasms, or breast cancer, nonhealing agnogenic vaginal anomalous bleeding, use of hormone therapy within 3 months prior to the trial, allergies, severe primary diseases of the cardiovascular system, cerebral vessels, liver, kidney, or hematopoietic system, and mental illnesses were excluded from this trial.

### 2.3. Randomization and Interventions

Participants were randomly assigned using SAS 6.1.2 software (SAS Institute, Inc., Cary, North Carolina) and stratified in the clinical centers (7 hospitals). Treatment assignments were sent to the DME (Design, Measurement, and Evaluation in Clinical Research) Center of Second Affiliated Hospital of Guangzhou University of Traditional Medicine, where medications, including Chinese herbal medicine (CHM) and placebo, were packaged, labeled with a sequential identification number, and sent to each clinical center. 

There were 4 study groups:

 
*PSY + CHM group*: patients in this group were treated with TCM-based psychotherapy and CHM (Gengnianningxin capsule or Bushen oral liquid for the patients with Kidney-Yin deficiency and Kidney-Yang deficiency, resp.);
 
*PSY group*: patients were treated with TCM-based psychotherapy and a corresponding CHM placebo (Gengnianningxin capsule placebo or Bushen oral liquid placebo) according to the corresponding TCM pattern;
 
*CHM group*: patients were treated with CHM (Gengnianningxin capsule or Bushen oral liquid for the patients with Kidney-Yin deficiency and Kidney-Yang deficiency, respectively);
 
*Placebo group*: patients were treated with a corresponding CHM placebo (Gengnianningxin capsule placebo or Bushen oral liquid placebo), according to the TCM pattern.

#### 2.3.1. Chinese Herbal Medicine Interventions

The Gengnianningxin capsules and Bushen oral liquid were authorized hospital herbal products in Guangdong Province Hospital of TCM. Gengnianningxin capsules, provided by Guizhou Xintian Pharmaceutical Co. Ltd. (Patch no. 020401), contain (in every 12 capsules) extracts of *Radix Rehmanniae preparata* (ShuDihuang) 15 g, *Rhizoma Coptidis* (Huanglian) 5 g, *Radix Paeoniae Alba *(Baishao) 9 g, *Radix Scutellariae Baicalensis* (Huangqin) 6 g, *Colla Corii Asini* (Ejiao) 12 g, and* poria* (Fuling) 10 g; they were administered to patients diagnosed with Kidney-Yin deficiency pattern, who were given 4 capsules per dose, 3 times per day following meals. 

Bushen oral liquid, provided by Guangdong Province Hospital of TCM (Patch no. 020405), contains extract of *Radix Rehmanniae preparata* (ShuDihuang) 10 g, *Fructus Ligustri Lucidui* (Nvzhenzi) 20 g, *Placenta Hominis* (Ziheche) 9 g, *Herba Epimedii Brevicornus* (Xianlingpi) 9 g, *Rhizoma Atractylodis Macrocephalae* (Baizhu) 9 g, *Rhizoma Alismatis* (Zexie) 12 g, *Concha Margaritifera Usta* (Zhenzhumu) 20 g, *Concha Ostreae* (Muli) 20 g, and *Rhizoma Chanxiong* (Chuanxiong) 6 g in every 2 bottles. It was used for the Kidney-Yang deficiency pattern; 1 bottle was given per dose, 2 times per day after meals. 

The two corresponding placebos were prepared to be identical in color, taste, and consistency to the 2 CHMs. The administration of placebos was performed in the same way as the corresponding CHMs. Both placebos were produced by Guangdong Hospital of TCM, Guangdong, China. 

All CHMs were provided to the patients free of charge. 

#### 2.3.2. Intervention of TCM-Based Psychotherapy

The facilities for psychotherapy included a bright, quiet, euthermic consulting room, which was equipped with luminaire, 6 to 10 soft, comfortable seats, DVD equipment, and equipment in case of emergency during psychotherapy. A physician with professional psychological expertise and technical skills for emergency management as well as a professional nurse with knowledge of both mental illness nursing and emergency procedures was involved in the psychotherapy. 

TCM psychotherapy procedures consisted of the following 4 steps.

**First Psycho-Communication**

During the first week, the physician should discuss psychological factors of the illness through in-depth one-on-one communication in the consulting room with each patient. No time limited in this step.

**Second Induction of the Catharsis**

Right after the first step, in the darkened psychotherapy room, the physician should induce a strong sad emotion, invoking tears in the patient by displaying a tragedy; according to TCM theory, this can be helpful to get rid of unhealthy feelings. The second step requires approximately 20 minutes.

**Third Induction of Positive Emotions**

At the second, Third and fifth weeks of the treatment, in the psychotherapy room (in bright light), the physician should induce happiness and laughter in the patient by showing them a comedy, which can balance the various emotions of the patient. This therapy requires 30 minutes per session once per week.

**Fourth Communication with Other Patients**

In the seventh and eleventh weeks, in a bright room with comfortable chairs, a nurse was in charge of a 30-minute symposium in which 10 to 20 patients took part. Free discussion and talk were encouraged regarding prevention, treatment, and nursing of menopausal syndrome. The monograph of every symposium was published and sent to each patient afterwards.

Patients participated in the above interventions for 3 months and underwent a followup observation for 3 more months. During the followup, only the menopausal symptoms and experimental examinations were recorded; no medical intervention was provided. 

For assurance of quality control, all researchers were well trained in the standard operating procedures (SOPs) for TCM psychotherapy procedure prior to beginning the clinical trial. The CHM and placebo conditions were blinded from both the patients and researchers.

### 2.4. Outcome and Measurements

The primary measurement of efficacy was the Kupperman Index (KI) [14] and the Menopause-Specific Quality of Life (MENQOL), which has four subscales (vasomotor, physical, psychosocial, and sexual) [15, 16]. The KI was obtained at weeks 4, 8, 12, 16, 20, and 24, while the MENQOL was measured at weeks 12 and 24. The primary outcome was changes in the KI and the total scores on subscales of the MENQOL from baseline to followup. In this case, negative values indicate an improving condition.

The KI is a numerical index used to determine the level of severity of menopause [14]. It consists of 11 items, including hot flushes, paraesthesia, insomnia, nervousness, melancholia, vertigo, weakness, arthralgia or myalgia, headache, palpitations, and formication. Each item is measured on a 0 to 3 point scale (where 0 = no symptoms and 3 = most severe), with symptoms weighed as follows: vasomotor symptoms by 4, nervousness, insomnia, and paraesthesia by 2, and other symptoms by 1. Total KI scores range from 0 to 51 points. A decrease in the total score denotes an improving condition.

The MENQOL questionnaire is a self-administered instrument that demonstrates potential for determining differences among menopausal women, both in quality of life and in changes in quality of life over time. It includes 29 questions in four domains [15]: vasomotor (Items 1, 2, and 3), psychosocial (Items 4–10), physical (Items 11–26), and sexual (Items 27–29). Each domain is scored separately, the detailed questionnaire is shown in Supplementary Material available online at doi:10.1155/2012/354145 [15, 17]. There is no overall score obtained from this questionnaire, as the relative contribution of each domain to the overall score is unknown. Subjects responded “No” to problems they did not experience and rated the symptoms that they did experience from 1 to 6 on a severity scale. Because the domain subscales are not composed of equal numbers of items, the mean of the subscale is used as the overall subscale score. For analyses, the domain scores are converted to a score system, each domain score ranges from 1 to 8. Decreases in total scores and domain scores represent an improving condition.

The secondary measurement of efficacy included serum levels of FSH and E2 and symptom relief. 

Safety evaluations included general physical examinations, routine blood, urine and stool samples, hepatic and renal function examinations, and type-B ultrasounds of the uterus, ovaries, and mammary glands. Additionally, any adverse events were reported at each clinic visit and recorded on a detailed form. 

### 2.5. Statistical Analysis

The sample size was determined based on the calculation of the effective rate of the placebo (61%) and the effective rate of psychotherapy plus CHM (85%). A sample size of 83 was calculated in each group using statistical methods [18–20]; when allowing for a withdrawal rate of 15%, the final optimal sample size was determined to be 98 in each group, with 392 participants in total. 

The primary outcomes of the study were comparisons of each treatment group against the placebo group; Dunnett's critical regions were adopted to maintain the overall *alpha* level of the study. 

Therapeutic effects, the differences between each treatment group and the placebo group with regards to the mean change from baseline, and the associated 95% confidence intervals (CIs) and *P* values were estimated using a multivariate mixed model (PROC MIXED in SAS). We used an unstructured covariance matrix for the repeated measures, as this structure best fit the data. Mixed models increase statistical power due to their ability to utilize followup data and to better handle missing data. Although retention rates were very high, a mixed-model analysis allowed us to use a true intention-to-treat approach, including data from all 424 randomly assigned women [21]. 

Mixed models were evaluated with adjustment for covariates. The adjusted models also controlled for age, body mass index (BMI), and menopausal status (current menopausal transition versus postmenopausal). All covariates except for BMI were selected a priori due to their hypothesized correlations with study outcomes and exposures. Bonferroni method was adopted for the *P* value adjustment in the multiple comparisons. 

Adverse event rates were compared between each treatment group and the placebo group using either chi-squared tests or the Fisher exact test (if the expected count was <5).

## 3. Results

**Participants and Followup**

In total, 920 cases diagnosed with menopausal syndrome were screened in the study; 424 eligible women participated in the trial and were randomly assigned as follows: PSY + CHM group (*n* = 105); PSY group (*n* = 104); CHM group (*n* = 111); placebo group (*n* = 104).

**Figure 1:**
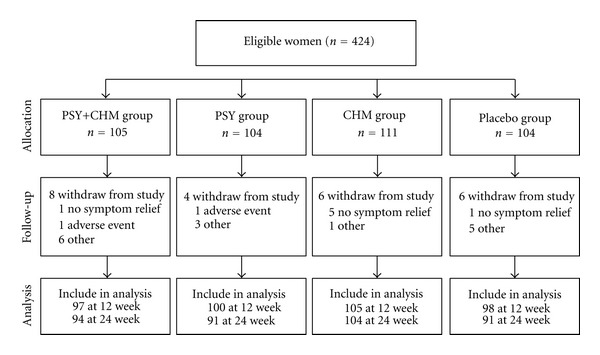
Participant recruitment, allocation, followup, and analysis.

Overall, 400 out of 424 participants completed 12 weeks of treatment, and 380 completed the KI and MENQOF at week 24 as shown in Figure 1. 

Baseline characteristics were similar among all groups, with the exceptions of menopausal statuses, Kupperman index scores and psychosocial subscale scores (Table 1). The ratios of menopausal transition (versus postmenopausal), KI score, and psychosocial score were lower in the CHM and placebo groups than in the PSY + CHM and PSY groups. 

### 3.1. Primary Outcomes

**Figure 2:**
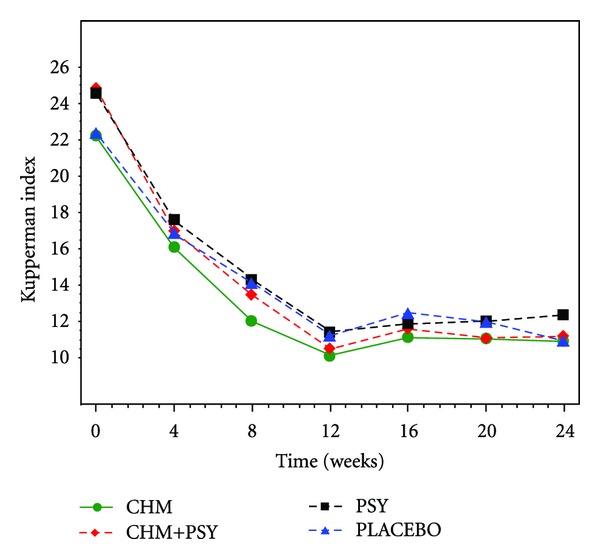
Adjusted mean number of Kupperman index score. The analysis was conducted on the data adjusted for age (continuous), body mass index (kg/m^2^, continuous), menopausal status (menopausal transition versus postmenopausal).

The average adjusted KI score (Figure 2) decreased between baseline and 12 weeks in all groups. There was a statistically significant difference in the average adjusted change between the PSY + CHM group and placebo group from 4 to 20 weeks, and a significant difference was found between the PSY group and placebo group at both 8 and 16 weeks. There were no significant differences between the patients treated with CHM and the placebo group (Table 2). According to overall time point measurements, there were differences between the PSY + CHM, PSY and placebo groups, as well as between the PSY + CHM and CHM groups. Yet after *P* value adjustment with Bonferroni method, significant difference can only be detected between the PSY + CHM group and placebo group at overall time point, although the average KI scores in each group indicated a better trend in effect of PSY group compared with placebo and CHM added-on groups compared with no CHM groups (PSY + CHM versus PSY alone or CHM alone versus placebo), no superiority of CHM alone can be clarified in this study. 

**Figure 3:**
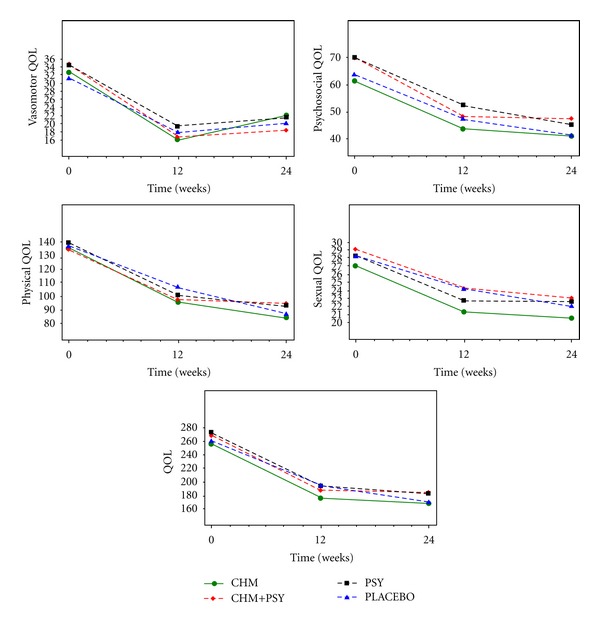
Adjusted mean number of MENQOL score and the scores of its subscales of vasomotor, psychosocial, physical, and sexual.

The MENQOL and subscale scores (Figure 3) decreased during the treatment and followup in all groups. However, after *P* value adjustment, there was no statistically significant differences in the average adjusted change in any subscale scores between any of the 2 groups at 12 and 24 weeks after the treatment and overall time points (Table 3).

### 3.2. Adverse Events and Adherence

There were no statistically significant differences between any of the 3 treatment groups and placebo group in the proportion of women with adverse events during treatment (Table 4). No severe adverse events occurred during the trial.

During the early 12-week treatment period, nearly all of the patients participated in treatment; a small number dropped out, resulting in an adherence rate of over 99.5% (Table 4). The primary reason for discontinuation of the treatment or study withdrawal was lack of symptom relief (*n* = 7) (Figure 1).

## 4. Discussion 

To our knowledge, this is the first randomized placebo-controlled clinical trial on efficacy evaluation of treating menopausal syndrome with combined TCM psychotherapy and CHM. The major finding is that TCM psychotherapy combined with CHM has a favorable outcome in treating menopausal syndrome; while single TCM psychotherapy or CHM was not statistically superior to placebo in this study. 

TCM has been involved in the Chinese healthcare system for thousands of years and plays an active role in both prevention of diseases and rehabilitation after recovery from severe illnesses [22]. Aimed at maintaining good health, TCM attempts to balance the body, mind, and spirit; thus, many treatment approaches are employed in this field, such as herbal medicine, acupuncture, massage, Taichi, cupping, and TCM psychotherapy [23]. TCM psychotherapy has been recorded in many ancient TCM books and has been successfully applied in the treatment of mental diseases or diseases with mental symptoms for thousands of years [24]. Distinct from modern psychotherapy, it has integrated TCM theory with several other therapeutic approaches, including psychological counseling and cognitive-behavioral techniques; it aims to harmonize the human body with mental and social statuses. This feature of TCM psychotherapy provides high applicability in the management of psychosomatic diseases, such as female menopausal syndrome [11, 24]. 

Though TCM psychotherapy has been increasingly accepted worldwide, the evidence for evaluating its efficacy remains nearly nonexistent [11]. Modern clinical studies, especially with randomized controlled trials (RCTs), usually ignore TCM psychotherapy due to the highly personalized therapeutic regimen and the difficulties of fulfilling the treatment. Therefore, a reasonable RCT that adheres to the principles of evidence-based medicine in obtaining concrete evidence for the efficacy of TCM psychotherapy is imperative; the key points involve the selection of a therapeutic regimen. In our present study, a clear-cut and easily performed therapeutic regimen is framed according to TCM psychotherapy principles with a definite SOP for practice. The 4 steps follow a regular routine of problem finding, solving, renewing, and consolidating. 

As a primary therapeutic approach, CHM has been successfully used in the treatment of female illnesses, applying skills gained from long-term clinical experience. A recent study demonstrated that CHM treatment has impacts on patients with infertility resulting from polycystic ovarian syndrome, anxiety, stress, and immunological disorders [25]. In this study, we tried to maximize the clinical effects by combining TCM psychotherapy with CHM therapy; psychotherapy plus placebo treatment, the single CHM treatment, and placebos were employed as control groups for obtaining an objective conclusion in this study. The change of KI in each group indicates that the combination of TCM psychotherapy and CHM is an effective treatment for menopausal syndrome compared with the placebo. Additionally, the combination therapy can significantly improve vasomotor symptoms compared with placebo. Moreover, the curative effect remains stable for at least 12 weeks following drug discontinuance, and the outstanding adherence and safety profile of these therapy regimens are additional advantages that can assure quality in clinical application on a larger scale. 

The single CHM or TCM psychotherapy therapy shows little advantage over the placebo in reducing the KI score and MENQOL scores in the present study. The efficacy of CHM for relieving the symptoms in menopausal women has been tested in animal [26–29] and clinical studies [30–32]; however, the results vary [21, 33]. There are many factors impacting the outcome of CHM or TCM psychotherapy in the treatment of menopausal syndrome, such as the selection of formulae and course of treatment, among others. Generally, there is a lack of long-term followup beyond the trial duration of 6–12 weeks, and well-designed, randomized controlled trials are needed to elucidate the true effects of these therapies beyond the placebo effect [34, 35]. Additionally, the insufficient manifestation of effectiveness in PSY and CHM alone group is possibly due to the inadequate sample size; after *P* value adjustment with Bonferroni method, significant difference can only be detected between the PSY + CHM group and placebo group, although the average KI scores in each group indicated a better trend of effect in PSY group compared with placebo group and CHM added-on groups compared with no CHM groups (PSY + CHM versus PSY alone or CHM alone vs placebo), thus future studies with larger sample size are still warranted to clarify the superiority of TCM based psychotherapy and CHM alone therapy for menopause syndrome. In this placebo-controlled clinical study, though the efficacy of CHM or TCM psychotherapy is poorly testified, the synergism of CHM and TCM psychotherapy is apparent. The mechanism of the effect enhancement of the combination therapy requires further examination in future studies. 

The major limitation of this study lies in the patient selection. Because only patients diagnosed with TCM patterns of *kidney deficiency* were recruited, not all menopausal syndrome patients were sampled; the efficacy of this combination therapy for global menopausal syndrome patients needs to be testified by expanding the trials. A further simplified and more easily understood procedure design should be another goal in future studies. 

## 5. Conclusions

The combination use of TCM psychotherapy and CHM can augment the effects, apparently reducing the KI score and the vasomotor score of the MENQOL, in the treatment of menopausal syndrome. 

## Supplementary Material

The MENQOL questionnaire is a self-administered instrument that demonstrates potential for determining differences among menopausal women, both in quality of life and in changes in quality of life over time. It includes 29 questions in four domains: vasomotor (Items 1, 2, and 3), psychosocial (Items 4–10), physical (Items 11–26), and sexual (Items 27–29). Each domain is scored separately, the detailed questionnaire is shown in Supplementary 1 file. There is no overall score obtained from this questionnaire, as the relative contribution of each domain to the overall score is unknown. Subjects responded “No” to problems they did not experience and rated the symptoms that they did experience from 1 to 6 on a severity scale. Because the domain subscales are not composed of equal numbers of items, the mean of the subscale is used as the overall subscale score. For analyses, the domain scores are converted to a score system, each domain score ranges from 1 to 8. Decreases in total scores and domain scores represent an improving condition.

## Figures and Tables

**Table 1:** Baseline on clinical and demographic characteristics in all groups (mean and standard deviations).

Characteristic	PSY + CHM (*n* = 104)	PSY (*n* = 105)	CHM (*n* = 111)	Placebo (*n* = 104)
Mean age (SD), y	49.77 (2.92)	50.16 (2.79)	49.5 (2.86)	49.68 (2.93)
Mean body mass index (SD), kg/m^2^	22.65 (2.29)	23.63 (3.21)	23.17 (2.76)	23.1 (2.81)
Menopausal transition (versus postmenopausal), *n* (%)	66 (62.9)	69 (66.4)	59 (53.2)	55 (52.9)
Mean Kupperman index score (SD)	24.85 (7.55)	24.58 (6.86)	22.21 (5.79)	22.38 (6.37)
Mean MENQOL score (SD) [12]	267.82 (73.72)	272.27 (64.42)	256.52 (70.28)	261.32 (69.93)
Mean vasomotor subscale score (SD)	34.7 (9.77)	34.39 (9.02)	32.67 (9.02)	31.26 (11.79)
Mean psychosocial subscale score (SD)	69.6 (24.22)	69.88 (22.51)	61.17 (24.86)	63.57 (25.88)
Mean physical subscale score (SD)	134.42 (47.22)	139.66 (44.33)	135.6 (42.92)	137.72 (40.6)
Mean sexual subscale score (SD)	29.1 (15.26)	28.33 (16.33)	27.08 (15.21)	28.37 (15.26)

**Table 2:** Difference in adjusted mean change of KI score between interventions and placebo Group*.

Time/weeks	CHM + PSY versus placebo			PSY versus placebo			CHM versus placebo			CHM + PSY versus PSY			CHM versus CHM + PSY			CHM versus PSY		
	Difference in mean change (95% CI)	*P* value	*P* adjust	Difference in mean change (95% CI)	*P* value	*P* adjust	Difference in mean change (95% CI)	*P* value	*P* adjust	Difference in mean change (95% CI)	*P* value	*P* adjust	Difference in mean change (95% CI)	*P* value	*P* adjust	Difference in mean change (95% CI)	*P* value	*P* adjust
4	−2.38	0.0109*	1	−1.49	0.1136	1	−0.64	0.4857	1	−0.89	0.3369	1	1.74	0.0567	1	0.84	0.3572	1
	(−5.88–1.12)			(−5–2.03)			(−4.09–2.81)			(−4.39–2.6)			(−1.68–5.16)			(−2.59–4.28)		
8	−3.08	0.0022*	0.6026	−1.99	0.0483*	1	−1.91	0.0532	1	−1.09	0.2751	1	1.17	0.2337	1	0.08	0.9373	1
	(−6.85–0.69)			(−5.76–1.78)			(−5.61–1.79)			(−4.85–2.66)			(−2.52–4.86)			(−3.62–3.77)		
12	−3.12	0.0034*	0.9401	−2	0.0598	1	−0.89	0.3927	1	−1.12	0.2928	1	2.23	0.0333*	1	1.11	0.2886	1
	(−7.11–0.87)			(−5.98–1.98)			(−4.8–3.02)			(−5.1–2.87)			(−1.69–6.14)			(−2.8–5.02)		
16	−3.27	0.0051*	1	−2.79	0.017*	1	−1.18	0.3011	1	−0.48	0.6808	1	2.09	0.0678	1	1.61	0.1605	1
	(−7.65–1.1)			(−7.18–1.59)			(−5.48–3.11)			(−4.86–3.9)			(−2.2–6.38)			(−2.69–5.91)		
20	−3.29	0.0046*	1	−2.17	0.0621	1	−0.74	0.5155	1	−1.12	0.3338	1	2.55	0.0248*	1	1.43	0.2089	1
	(−7.63–1.06)			(−6.52–2.19)			(−5–3.52)			(−5.47–3.23)			(−1.7–6.8)			(−2.83–5.68)		
24	−2.11	0.0585	1	−0.69	0.535	1	0.18	0.8676	1	−1.42	0.2041	1	2.3	0.0354*	1	0.88	0.4227	1
	(−6.3–2.07)			(−4.89–3.5)			(−3.92–4.28)			(−5.61–2.77)			(−1.79–6.39)			(−3.22–4.98)		
Over all time points	−2.88	0.0021*	0.0126*	−1.85	0.0476*	0.2853	−0.86	0.3463	1	−1.02	0.2736	1	2.01	0.0279*	0.1672	0.99	0.2797	1
	(−5.34–0.41)			(−4.32–0.62)			(−3.29–1.56)			(−3.48–1.44)			(−0.4–4.43)			(−1.43–3.41)		

*P < 0.05.

*All analyses were conducted on the data adjusted for age (continuous), body mass index (kg/m^2^, continuous), menopausal status (menopause transition versus postmenopausal). Estimates of difference in mean change from baseline versus placebo, along with *P* values and 95% CIs, from mixed-model analysis that used data from baseline and all follow-up time points (total, *n* = 424 for adjusted analyses). Bonferroni method was adopted for the *P* value adjustment in the multiple comparisons.

**Table 3:** Difference in adjusted mean change of MENQOL and its subscales score between interventions and placebo group*.

Subscales	Time/weeks	PSY + CHM versus Placebo			PSY versus Placebo			CHM versus Placebo			CHM + PSY versus PSY			CHM versus CHM + PSY			CHM versus PSY		
		Difference in mean change (95% CI)	*P* value	*P* adjust	Difference in mean change (95% CI)	*P* value	*P* adjust	Difference in mean change (95% CI)	*P* value	*P* adjust	Difference in mean change (95% CI)	*P* value	*P* adjust	Difference in mean change (95% CI)	*P* value	*P* adjust	Difference in mean change (95% CI)	*P* value	*P* adjust
	12	−1.08	0.6044	1	1.49	0.4704	1	−1.89	0.3551	1	−2.57	0.2171	1	−0.81	0.6933	1	−3.39	0.0982	1
		(−8.14–5.98)			(−5.52–8.51)			(−8.82–5.04)			(−9.64–4.49)			(−7.79–6.17)			(−10.32–3.54)		
Vasomotor	24	−1.74	0.4188	1	1.32	0.5413	1	1.92	0.3625	1	−3.06	0.1561	1	3.66	0.0816	1	0.59	0.7780	1
		(−9.04–5.56)			(−6.01–8.66)			(−5.21–9.05)			(−10.37–4.25)			(−3.45–10.76)			(−6.55–7.74)		
	Over all time points	0.21	0.8851	1	1.98	0.1628	0.9771	0.48	0.7322	1	−1.78	0.2114	1	0.27	0.8456	1	−1.51	0.2808	1
		(−3.56–3.97)			(−1.78–5.74)			(−3.22–4.17)			(−5.54–1.99)			(−3.43–3.97)			(−5.2–2.19)		

	12	0.95	0.7936	1	4.85	0.1825	1	−3.57	0.3188	1	−3.89	0.2860	1	−4.53	0.2086	1	−8.42	0.0192*	1
		(−11.41–13.32)			(−7.47–17.17)			(−15.73–8.58)			(−16.26–8.47)			(−16.73–7.67)			(−20.58–3.73)		
Psychosocial	24	6.06	0.0992	1	3.88	0.2919	1	−0.24	0.9471	1	2.18	0.5527	1	−6.3	0.0799	1	−4.12	0.2530	1
		(−6.38–18.5)			(−8.59–16.34)			(−12.42–11.94)			(−10.27–14.64)			(−18.47–5.87)			(−16.31–8.08)		
	Over all time points	4.35	0.1517	0.9103	5.01	0.0984	0.5907	−2.07	0.4876	1	−0.67	0.8261	1	−6.42	0.0318	0.1910	−7.08	0.0179	0.1073
		(−3.68–12.38)			(−3.01–13.04)			(−9.97–5.83)			(−8.69–7.36)			(−14.32–1.48)			(−14.98–0.81)		

	12	−8.63	0.1967	1	−5.65	0.3962	1	−11.03	0.0936	1	−2.98	0.6552	1	−2.39	0.7163	1	−5.38	0.4129	1
		(−31.28–14.02)			(−28.21–16.91)			(−33.28–11.23)			(−25.63–19.66)			(−24.74–19.95)			(−27.63–16.88)		
Physical	24	7.73	0.2595	1	5.57	0.4184	1	−3	0.6542	1	2.17	0.7523	1	−10.74	0.1092	1	−8.57	0.2025	1
		(−15.5–30.96)			(−17.75–28.88)			(−25.74–19.73)			(−21.1–25.43)			(−33.43–11.96)			(−31.35–14.21)		
	Over all time points	−1.4	0.7987	1	0.62	0.9101	1	−5.38	0.3193	1	−2.02	0.713	1	−3.98	0.4611	1	−6	0.2669	1
		(−15.94–13.14)			(−13.93–15.17)			(−19.69–8.93)			(−16.57–12.53)			(−18.29–10.33)			(−20.31–8.31)		

	12	0.14	0.9489	1	−1.46	0.5146	1	−2.88	0.1921	1	1.6	0.4736	1	−3.02	0.1704	1	−1.42	0.5181	1
		(−7.46–7.75)			(−9.05–6.13)			(−10.36–4.6)			(−5.98–9.19)			(−10.49–4.45)			(−8.87–6.03)		
Sexual	24	1.02	0.6568	1	0.61	0.7915	1	−1.45	0.5175	1	0.41	0.8578	1	−2.47	0.2683	1	−2.06	0.3578	1
		(−6.74–8.78)			(−7.18–8.4)			(−9.05–6.15)			(−7.34–8.15)			(−10.02–5.09)			(−9.65–5.53)		
	Over all time points	0.63	0.7404	1	−0.3	0.8753	1	−1.87	0.3176	1	0.93	0.6239	1	−2.51	0.1795	1	−1.58	0.399	1
		(−4.41–5.68)			(−5.35–4.75)			(−6.84–3.09)			(−4.09–5.96)			(−7.44–2.43)			(−6.52–3.37)		

	12	−8.18	0.4723	1	−1.1	0.9225	1	−18.85	0.0924	1	−7.08	0.5327	1	−10.67	0.3397	1	−17.74	0.1118	1
		(−46.75–30.39)			(−39.59–37.38)			(−56.76–19.06)			(−45.52–31.37)			(−48.54–27.2)			(−55.52–20.04)		
QOL	24	13.88	0.2295	1	11.58	0.3175	1	−2.08	0.8540	1	2.3	0.8415	1	−15.96	0.1565	1	−13.66	0.2266	1
		(−25.25–53.02)			(−27.67–50.82)			(−40.42–36.26)			(−36.76–41.37)			(−54.12–22.19)			(−51.93–24.61)		
	Over all time points	4.07	0.6629	1	7.14	0.4444	1	−8.58	0.3502	1	−3.07	0.7408	1	−12.64	0.1668	1	−15.72	0.0861	0.5165
		(−20.64–28.77)			(−17.58–31.86)			(−32.89–15.73)			(−27.69–21.54)			(−36.84–11.56)			(−39.93–8.5)		

^∗^ *P* < 0.05.

^∗^All analyses were conducted on the data adjusted for age (continuous), body mass index (kg/m^2^, continuous), menopausal status (menopause transition versus postmenopausal). Estimates of difference in mean change from baseline versus placebo, along with *P* values and 95% CIs, from mixed-model analysis that used data from baseline and all follow-up time points (total, *n* = 424 for adjusted analyses).

**Table 4:** Women with adverse events, mean adherence by treatment group over 24 weeks of follow-up.

Variable	CHM (*n* = 111)	PSY + CHM (*n* = 105)	PSY (*n* = 104)	Placebo (*n* = 104)
Adverse events				
Oppression in the chest, *n* (%)	—	2 (1.92)	1 (0.95)	—
Other, *n* (%) (sore throat, abdominal distension, distending pain in chest, stomachache, oral ulcer, skin rash and nocturia)	3 (2.70)	6 (5.77)	2 (1.90)	3 (2.88)
Adherence				
Average medications taken, %	99.92	99.83	100.00	99.75
Reasons for withdrawal, *n* (%)				
No symptom relief	5 (4.50)	1 (0.96)	—	1 (0.96)
Adverse events	—	1 (0.96)	1 (0.95)	—
Other	1 (0.90)	6 (5.77)	3 (2.86)	5 (4.81)

## References

[1] Avis NE, Brockwell S, Colvin A (2005). A universal menopausal syndrome?. *American Journal of Medicine*.

[2] Brenner PF (198). The menopausal syndrome. *Obstetrics & Gynecology*.

[3] McKinlay SM, Jefferys M (1974). The menopausal syndrome. *British Journal of Preventive and Social Medicine*.

[4] Hunter M, Battersby R, Whitehead M (1986). Relationships between psychological symptoms, somatic complaints and menopausal status. *Maturitas*.

[5] Coope J (1996). Hormonal and non-hormonal interventions for menopausal symptoms. *Maturitas*.

[6] Hunter MS (1993). Predictors of menopausal symptoms: psychosocial aspects. *Bailliere’s Clinical Endocrinology and Metabolism*.

[7] Ayers B, Forshaw M, Hunter MS (2010). The impact of attitudes towards the menopause on women’s symptom experience: a systematic review. *Maturitas*.

[8] Yen JY, Yang MS, Wang MH, Lai CY, Fang MS (2009). The associations between menopausal syndrome and depression during pre-, peri-, and postmenopausal period among Taiwanese female aborigines. *Psychiatry and Clinical Neurosciences*.

[9] García CL, Gómez-Calcerrada SG (2011). Cognitive-behavioral intervention among women with slight menopausal symptoms: a pilot study. *Spanish Journal of Psychology*.

[10] Qian LQ, Wang B, Niu JY, Gao S, Zhao DY (2010). Assessment of the clinical effect of Chinese medicine therapy combined with psychological intervention for treatment of patients of peri-menopausal syndrome complicated with hyperlipidemia. *Chinese Journal of Integrative Medicine*.

[11] Ying J, Bowei W (2011). Wang xiao-yun using emotion-thought therapy of Chinese medicine to treat neuropsychological symptoms of menopausal syndrome. *China Journal of Chinese Medicine*.

[12] Kilic S, Yilmaz N, Erdogan G (2010). Effect of non-oral estrogen on risk markers for metabolic syndrome in early surgically menopausal women. *Climacteric*.

[13] Ministry of Health of The People’s Republic of China (1997). *Guidelines on Clinical Research of Treating Menopausal Syndrome with Chinese Medicine*.

[14] Kupperman HS, Wetchler BB, Blatt MH (1959). Contemporary therapy of the menopausal syndrome. *The Journal of the American Medical Association*.

[15] Hilditch JR, Lewis J, Peter A (1996). A menopause-specific quality of life questionnaire: development and psychometric properties. *Maturitas*.

[16] Hilditch JR, Lewis J, Peter A (1996). A menopause-specific quality of life questionnaire: development and psychometric properties. *Maturitas*.

[17] Popivanov P (1983). Menopausal indices as criteria for the effectiveness of acupuncture treatment of the climacteric syndrome (preliminary report). *V*ŭ*treshni Bolesti*.

[18] Hauer-Jensen M (1993). Sample size calculation, power analysis and randomization: research project design in Windows. *Computer Applications in the Biosciences*.

[19] Gould AL (1993). Sample sizes for event rate equivalence trials using prior information. *Statistics in Medicine*.

[20] Kay R (1998). Statistical principles for clinical trials. *Journal of International Medical Research*.

[21] Newton KM, Reed SD, LaCroix AZ, Grothaus LC, Ehrlich K, Guiltinan J (2006). Treatment of vasomotor symptoms of menopause with black cohosh, multibotanicals, soy, hormone therapy, or placebo: a randomized trial. *Annals of Internal Medicine*.

[22] Jiang M, Zhang C, Cao H, Chan K, Lu A (2011). The role of Chinese medicine in the treatment of chronic diseases in China. *Planta Medica*.

[23] Barnes LL (1998). The psychologizing of Chinese healing practices in the United States. *Culture, Medicine and Psychiatry*.

[24] Jie Z, Yanling D (2009). TCM-based Psychotherapy and the mechanism. *Lishizhen Medicine and Materia Medica Research*.

[25] Huang ST, Chen AP (2008). Traditional Chinese medicine and infertility. *Current Opinion in Obstetrics and Gynecology*.

[26] Lee SC, Chang SJ, Tsai LY (2004). Effects of traditional Chinese medicines on serum lipid profiles and homocysteine in the ovariectomized rats. *American Journal of Chinese Medicine*.

[27] Li H, Li SL, Gong L, Wang JL, Li YZ, Wu ZH (2008). The effects of an herbal medicine Bu-Wang-San on learning and memory of ovariectomized female rat. *Journal of Ethnopharmacology*.

[28] Li H, Li SL, Wu ZH, Gong L, Wang JL, Li YZ (2009). Effect of traditional Chinese herbal Bu-Wang-San on synaptic plasticity in ovariectomised rats. *Journal of Pharmacy and Pharmacology*.

[29] Li JJ, Li JT, Fu JP (2007). Erxian tang—introduction of a Chinese herbal formula, clinical practice, and experimental studies. *Chinese Journal of Integrative Medicine*.

[30] Kwee SH, Tan HH, Marsman A, Wauters C (2007). The effect of Chinese herbal medicines (CHM) on menopausal symptoms compared to hormone replacement therapy (HRT) and placebo. *Maturitas*.

[31] Liu D, Lu Y, Ma H (2009). A pilot observational study to assess the safety and efficacy of menoprogen for the management of menopausal symptoms in Chinese women. *Journal of Alternative and Complementary Medicine*.

[32] Noguchi M, Yuzurihara M, Ikarashi Y (2009). Effects of the traditional Japanese medicine Tokaku-jyoki-to in rat-models for menopausal hot flash. *Journal of Ethnopharmacology*.

[33] Chen LC, Wang BR, Chen IC, Shao CH (2010). Use of Chinese herbal medicine among menopausal women in Taiwan. *International Journal of Gynecology and Obstetrics*.

[34] Wong VCK, Lim CED, Luo X, Wong WSF (2009). Current alternative and complementary therapies used in menopause. *Gynecological Endocrinology*.

[35] Kupferer EM, Dormire SL, Becker H (2009). Complementary and alternative medicine use for vasomotor symptoms among women who have discontinued hormone therapy. *Journal of Obstetric, Gynecologic, and Neonatal Nursing*.

