# Supplementary material for: Effect of Combining Therapy with Traditional Chinese Medicine-Based Psychotherapy and Herbal Medicines in Women with Menopausal Syndrome: A Randomized Controlled Clinical Trial
Source: Evid Based Complement Alternat Med. 2012 Dec 10;2012:354145. doi: 10.1155/2012/354145 (PMC3523610; doi:10.1155/2012/354145)
Supplement: Supplementary file 1 — The MENQOL questionnaire is a self-administered instrument that demonstrates potential for determining differences among menopausal women, both in quality of life and in changes in quality of life over time. It includes 29 questions in four domains: vasomotor (Items 1, 2, and 3), psychosocial (Items 4–10), physical (Items 11–26), and sexual (Items 27–29). Each domain is scored separately, the detailed questionnaire is shown in Supplementary 1 file. There is no overall score obtained from this questionnaire, as the relative contribution of each domain to the overall score is unknown. Subjects responded “No” to problems they did not experience and rated the symptoms that they did experience from 1 to 6 on a severity scale. Because the domain subscales are not composed of equal numbers of items, the mean of the subscale is used as the overall subscale score. For analyses, the domain scores are converted to a score system, each domain score ranges from 1 to 8. Decreases in total scores and domain scores represent an improving condition. [file 354145.f1.docx]

**Supplementary file:**

MENQOL questionnaire contains 29 items as follow: 1) Hot flushes or flashes; 2) Night sweats; 3) Sweating; 4) Being dissatisfied with my personal life; 5) Feeling anxious or nervous; 6) Experiencing poor memory; 7) Accomplishing less than I used to; 8) Feeling depressed, down or blue; 9) Being impatient with other people; 10) Feelings of wanting to be alone; 11) Flatulence (wind) or gas pains; 12) Aching in muscles and joints; 13) Feeling tired or worn out; 14) Difficulty sleeping; 15) Aches in back of neck or head; 16) Decrease in physical strength; 17) Decrease in stamina; 18) Feeling a lack of energy; 19) Drying skin; 20) Weight gain; 21) Increased facial hair; 22) Changes in appearance, texture or tone of your skin; 23) Feeling bloated; 24) Low backache; 25) Frequent urination; 26) Involuntary urination when laughing or coughing; 27) Change in your sexual desire; 28) Vaginal dryness during intercourse; 29) Avoiding intimacy.
